# Harvesting of freshwater microalgae with microbial bioflocculant: a pilot-scale study

**DOI:** 10.1186/s13068-016-0458-5

**Published:** 2016-02-27

**Authors:** Theoneste Ndikubwimana, Xianhai Zeng, Theophile Murwanashyaka, Emmanuel Manirafasha, Ning He, Wenyao Shao, Yinghua Lu

**Affiliations:** Department of Chemical and Biochemical Engineering, College of Chemistry and Chemical Engineering, Xiamen University, 361005 Xiamen, China; College of Energy, Xiamen University, 361005 Xiamen, China; The Key Laboratory for Synthetic Biotechnology of Xiamen City, Xiamen University, 361005 Xiamen, China

**Keywords:** Microalgae, Pilot scale, In situ flocculation, Bioflocculant, Biochemical composition

## Abstract

**Background:**

Nowadays, bioflocculation is considered as a potential technology that could be able to alleviate microalgae dewatering cost regarded as the cornerstone hindrance of their full-scale application. However, most bioflocculation studies reported are laboratory scales. This study examined a pilot-scale and in situ flocculation of freshwater microalgae *Desmodesmus brasiliensis* by microbial bioflocculant. Biochemical composition of microalgal biomass was analyzed to evaluate the applicability of bioflocculation for microalgae-based biofuel production.

**Results:**

The flocculation efficiency >98 % was achieved at both pilot-scale and in situ treatment. Bioflocculation is simple, effective, economic, and environmentally friendly. Even though total proteins recovered from biomass harvested by centrifugation and that harvested by bioflocculation were significantly different, there was no significant difference in total carbohydrates and total lipids recovered from either biomass harvested by centrifugation or biomass harvested by bioflocculation.

**Conclusion:**

The results herein presented, doubtlessly demonstrated that the γ-PGA bioflocculant produced by *Bacillus licheniformis* CGMCC 2876 is applicable for commercial-scale microalgae harvesting. In addition, bioflocculation process cost could greatly be reduced by in situ operation as no investment cost is needed for a separate flocculation tank and mixing device. Furthermore, bioflocculation method developed is a worthy microalgae harvesting method for algal-based biofuel production.

**Graphical abstract:**
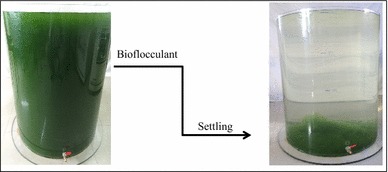
The addition of bioflocculant to microalgae cultures followed by mixing elicits, the formation of heavy flocs which settle out by gravity sedimentation in a relatively short settling time.

**Electronic supplementary material:**

The online version of this article (doi:10.1186/s13068-016-0458-5) contains supplementary material, which is available to authorized users.

## Background

Algal biomass is considered as the most assuring raw material to counterbalance the unremitting global demand for food, feed, and biofuel and chemical production [1, 2]. Microalgae have appreciable growth rate, high lipids, and carbohydrate yield and other biochemicals such as proteins and vitamins, also microalgae cultivation can be incorporated in different environmental bioremediation schemes [3–5]. However, regardless of these advantages, the major challenge lies in the dewatering of the microalgae cultures due to their high dilution rate, minor cell dimensions, and electronegative cell surface charge [6].

The concentrating reactions of algal biomass are particularly sensitive to pH, properties of the cellular surface, concentrations of the flocculants, and ionic strength of the culture solution [1, 7, 8], thus the most dewatering methods currently available are obstructed by either economic or technical drawbacks. It is known that by varying the initial microalgae culture pH acts upon the membrane surface charge of the microalgal cells and the ionic forms of dissolved salts available in the culture suspension will be modified [9]. For example, flocculation induced by pH increase is ascribable to precipitation of CaCO_3_, Mg(OH)_2_, and calcium phosphate [9], while the flocculation at decreased pH is due to charge neutralization as the carboxylate ions of organic matters attached to microalgal cells accept protons as a result of pH reduction [10].

Most of the solid–liquid separation methods applied for microalgae suspensions dewatering are likely applied to lab-scale conditions, and would issue severe challenges such as high energy consumption, long processing times, low recovery, and high greenhouse gas emissions, once applied at large-scale conditions [11].

Lately, naturally occurring microbial flocculants have been used to harvest microalgae for aquaculture and biodiesel production because of their high harvesting efficiency, and biodegradability [12, 13].

Bioflocculation is believed to address substantially dewatering cost since little or no energy consumption is required compared to centrifugation mostly applied in industry [14]. Moreover, much less capital and maintenance costs are incurred [7], and microalgae dewatering by bioflocculation has achieved significant efficiencies [7, 15–18]. Furthermore, bioflocculation is an innovative dewatering method, and environmentally friendly. Bioflocculation is a natural flocculation process hastened with biomolecules from microbial cells [14].

Recently, an innovative, economic, and environmentally friendly microalgae dewatering applying bacterial broth bioflocculant produced by *Bacillus licheniformis* CGMCC 2876 (containing active constituent of poly γ-glutamic acid, γ-PGA) was reported [7], with the mechanism governing this bioflocculation process [19]. High flocculation of 95 % for marine microalgae *Chlorella vulgaris* and freshwater microalgae *C. protothecoides* was reported by inducing flocculation with commercial γ-PGA bioflocculant produced by *B. subtilis* [18]. Furthermore, more than 98 % microalgae cells of *C. vulgaris* were entrapped in the fungal clumps as a result of co-cultivation of microalgae *C. vulgaris* with filamentous fungi [16, 17]. Although this harvesting method seemed promising, the increase in biomass was not proportional to lipid yield due to the decrease in culture pH. However, to our knowledge, no report was published about the microalgae dewatering by bioflocculation using bacterial bioflocculant at large scale.

This study is aimed to evaluate the scalability of the bioflocculation method for microalgae harvesting using bacterial broth bioflocculant produced by *B. licheniformis* CGMCC 2876, and the effectiveness of in situ treatment by the same method. The effect of bioflocculation technology on biochemical composition of microalgae biomass is investigated and presented.

## Results

### Flocculation character of microalgae cultures

Significant flocculation efficiencies were achieved by only changing the initial culture pH [9, 10]. In addition, higher or lower bioflocculant dosage may decrease the flocculation efficiency [7, 18]. Moreover, flocculation process is significantly affected by mixing. Effective mixing is necessary to alleviate the dispersion of the flocculant within the particles in as short period as possible, thus granting to obtain a uniform mixture of the flocculant and suspended cells to maximize effective destabilization of colloidal particles in order to initiate flocculation. After flash mixing, it is important to averagely mix the microalgae cultures so as to step-up the contact between flocculating particles and to facilitate the growth of large flocs [7]. The settling time depends on the floc size and it has been pointed out that when the flocs settle faster, the quality of particle removal is better. Experimental results have demonstrated that the addition of the flocculating agents followed by flash mixing and slow mixing allowed better interactions and flocs growth thus enhancing the flocculation performance [7, 20]. Therefore, it is imperative to optimize the operating parameters prior to scale up the flocculation process.

**Fig. 1:**
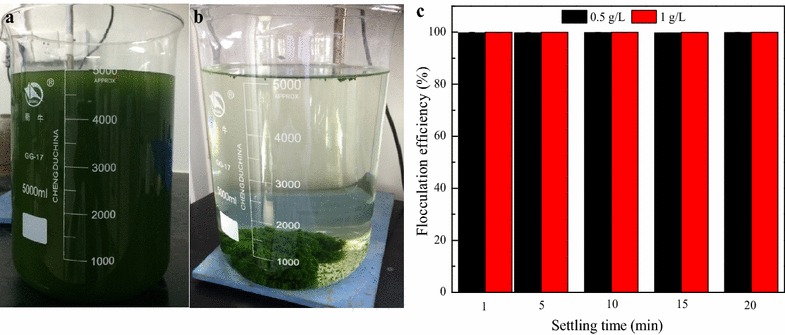
Bioflocculation of 5 L cultures of *D. brasiliensis* with the broth of *B. licheniformis* CGMCC 2876. **a** Microalgae cultures; **b** Flocculated microalgae; **c** Flocculation efficiency at different sedimentation times; *data* presented are the mean values of two independent replicates, and the *bars* represent the standard error of two replicates

In an attempt to scale up bioflocculation process for the freshwater microalgae *Desmodesmus brasiliensis*, firstly 5 L of microalgal cultures (biomass concentration: 0.5 and 1 g/L) were flocculated at operating conditions of 2.5 mL/L bioflocculant, 200 rpm mixing rate for 2 min at pH 3. The results are presented in Fig. 1.

The microalgal suspensions are seen with a dark-green color before flocculation (Fig. 1a) while flocs can be visualized settled at the bottom of the flocculation vessel after flocculation (Fig. 1b). The flocculation efficiency above 99 % was achieved after only 1 min of settling time either at 0.5 or 1 g/L of biomass concentration (Fig. 1c), the same flocculation rate was maintained after 20 min of settling time. These results are in agreement with small-scale results at which no significant differences were observed in flocculation efficiencies within the range of biomass concentration of 0.5–1.5 g/L [7]. Therefore, in further experiments, 0.5 g/L of biomass concentration was used.

**Fig. 2:**
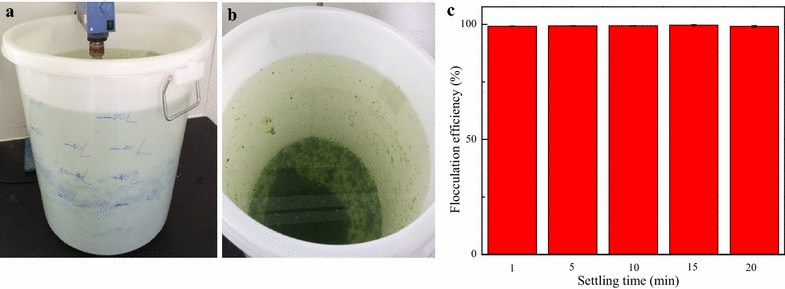
Bioflocculation of 50 L cultures of *D. brasiliensis* with the broth of *B. licheniformis* CGMCC 2876. **a** Microalgae cultures; **b** Flocculated microalgae; **c** Flocculation efficiency at different sedimentation times; *data* presented are the mean values of two independent replicates, and the *bars* represent the standard error of two replicates)

After bioflocculation of 5 L of microalgae cultures, 50 L was also flocculated following the same procedures as described for flocculation of 5 L microalgae cultures. It can be seen from the results depicted in Fig. 2a that microalgal suspensions were evenly distributed in flocculation vessel while a deposit of dark green (flocs) is discernible at the bottom of the flocculation vessel (Fig. 2b). A flocculation efficiency of 99.2 ± 0.2 % was achieved after only 1 min of settling time (Fig. 2c), and more than 98 % flocculation efficiency was maintained after a long period of sedimentation time.

**Fig. 3:**
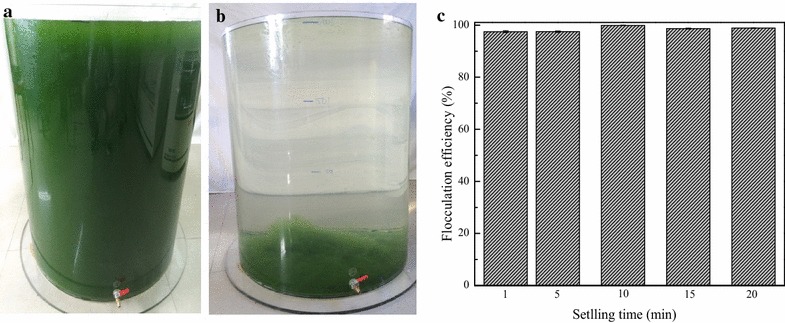
Bioflocculation of 200 L cultures of *D. brasiliensis* with the broth of *B. licheniformis* CGMCC 2876. **a** Microalgae cultures; **b** Flocculated microalgae; **c** Flocculation efficiency at different sedimentation times; *data* presented are the mean values of two independent replicates, and the *bars* represent the standard error of two replicates

Further scale up was conducted with 200 L of microalgae cultures, and the operating parameters were the same as described above. The results presented in Fig. 3a, b show the visual characteristics of the cultures before and after flocculation, respectively. The dark-green color observable in Fig. 3a disappeared in Fig. 3b with dense algal biomass (flocs) at the bottom of the flocculation tank. From this view, high flocculation efficiency is expected, and this hypothesis is evidenced by the experimental results depicted in Fig. 3c. The flocculation efficiency of 97.5 ± 0.4 % was achieved after only 1 min of settling time, and after 10 min of settling time, flocculation efficiency above 99 % was achieved. These results demonstrate that this novel technology of harvesting freshwater microalgae by γ-PGA broth bioflocculant is effective.

**Fig. 4:**
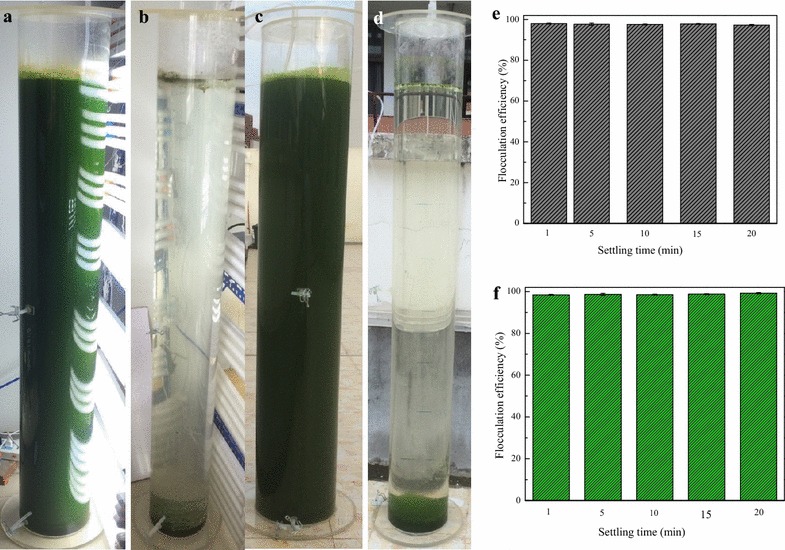
In situ bioflocculation of *D. brasiliensis* with the broth of *B. licheniformis* CGMCC 2876. **a**, **c** Microalgae cultures; **b**, **d** Flocculated microalgae; **e**, **f** Flocculation efficiency at different sedimentation times; *data* presented are the mean values of two independent replicates, and the *bars* represent the standard error of two replicates

In situ flocculation experiments produced the results presented in Fig. 4. The evenly dispersed microalgal cultures with dark-green color observable in Fig. 4a, c formed an algal biomass paste (flocs) at the bottom of the photobioreactor (PBR) (Fig. 4b, d). After only one min of settling time, a flocculation efficiency >98 % was achieved and the flocculation efficiency above 99 % was achieved after 20 min of settling time for both indoor and outdoor cultures treatment (Fig. 4e, f). These results demonstrate that in situ bioflocculation of freshwater microalgae *D. brasiliensis*is is feasible, however further studies are necessary to find optimum aeration rate required for effective mixing.

### Microalgae biomass characterization

Quantification of total carbohydrates, total proteins, and total lipids conducted as described in “Methods” generated the results presented in Table 1.

**Table 1:** Microalgal biomass biochemical composition

Harvesting method	Total carbohydrates (%)	Total proteins (%)	Total lipids (%)
Centrifugation	48.9 ± 3.4	18.4 ± 0	24.1 ± 2.5
Bioflocculation	44.25 ± 2.3	21.1 ± 0.42	24.2 ± 0.7

The table presents the composition in total percentage of carbohydrates, proteins, and lipids of microalgae *D. brasiliensis*

One can see from Table 1 that there is no significant difference in total carbohydrates and total lipids recovered from either biomass harvested by centrifugation or those recovered from biomass harvested by bioflocculation (*p* = 0.251, *p* = 0.981 for total carbohydrates and total lipids, respectively, *α* = 0.05). However, total proteins recovered from biomass harvested by centrifugation and that harvested by bioflocculation were significantly different (*p* = 0.001, *α* = 0.05).

As for the quantity of biochemicals recovered, there may be some variations due to the extraction method used. For example, the Phenol–Sulfuric Acid method used for total carbohydrates is notoriously variable, also it is known that all sugars do not exhibit a similar colorimetric response, therefore there could be over or underestimation of total carbohydrates if the calibration curve is conducted based on one neutral sugar [21]. The completeness of extraction and composition of total lipids depends largely on the biology of the algal species, the compatibility of the solvent polarity with the lipid molecules polarity and extraction conditions applied. Unavoidably the extractable oil fraction will contain amounts of chlorophyll, pigments, proteins, or soluble carbohydrates. Although gravimetric is the fast way to obtain total lipid data, the solvent system utilized will influence the gravimetric yield and composition of total lipids [22, 23]. If accurate data are required due to specific desired product or the objective of the microalgae study, alternative quantification methods are recommended. High-performance liquid chromatography (HPLC), Anion exchange chromatography (HPAEC) and Gas chromatography (GC) are recommended for identification and quantification of monomers after sequent hydrolysis of carbohydrate polymers in microalgae [21, 24]. The GC is recommended for quantification of the fatty acid methyl esters after an acid-catalyzed transesterification of extracted oils [22, 25, 26]. Furthermore, the most reliable method to quantify the total proteins recommended is the determination of amino acid composition by HPLC analysis [21].

Bioflocculation process utilizing the bioflocculant γ-PGA produced by *B. licheniformis* CGMCC 2876 herein presented achieve higher efficiency compared to other pilot or large-scale microalgae harvesting methods reported (Table 2). Furthermore, no significant differences found in biochemicals (e.g. carbohydrates and lipids) recovered from biomass harvested by either bioflocculation or centrifugation.

**Table 2:** Microalgae harvesting capableness of different technologies at pilot or large-scale conditions

Harvesting techniques	Microalgal species	Treated volume (L)	Harvesting efficiency (%)	References
Flocculation with *B. licheniformis* broth	*Desmodesmus brasiliensis*	50–200	≥98	This study
pH increment	*Tetraselmis suecica, Chaetoceros calcitrans, Chlorella muelleri, Skeletonema* sp.*, Rhodomonas salina, Attheya septentrionalis, Nitzschia closterium, Chlorella muelleri, Thalassiosira pseudonana*	10–1000	≥80	[28]
Foam fractionation	*Chaetoceros* sp.	220	90	[41]
Foam flotation	*Chlorella* sp.	10–10.2	≥92	[27, 54]
Flocculation	*Scenedesmus* sp.	1000	>96	[38]
Gravity sedimentation coupled with filtration	*Staurosira* sp., *Desmodesmus* sp.	200	80	[37]
Centrifugation	*Chaetoceros muelleri*	550	90	[40]
Flotation under vacuum	*Not precised*	2000	49.5	[39]

The table presents reported data about the efficiency of different technologies applied in microalgae harvesting at pilot or large-scale conditions in order to compare with the experimental results described in the present article

## Discussion

Effectiveness of different dewatering technologies at small-scale level have been extensively reported, however their scale up is questionable due to the severe problems they may elicit once applied at large-scale conditions [11]. Moreover, different methods currently applied for microalgae dewatering, although they could achieve significant efficiency, still are not economically workable especially for the low value products such as biofuels [11, 27]. Centrifugation is fast and can be applied successfully for microalgae dewatering, however cells are exposed to high risk of cell disruption and structural damage due to high gravitational and shear forces, also the method is energy consuming, thus the application of centrifugation for dewatering large quantities of microalgae culture is practically considered unsuitable [28, 29]. Different means of flocculation have been reported for their effective microalgae dewatering [28, 30–33], however dosages of chemical flocculants which affect the product quality and that require additional separation steps from the final product, construct the hindrance for industrial application of flocculation in microalgae dewatering. The addition of surfactants or collectors to improve flotation processes result in the same challenges as chemical flocculants. Electrolytic methods are believed to be cost effective and environmentally friendly microalgae dewatering methods and different experimental reports have confirmed the assumption [34, 35], nevertheless regular replacement of electrode materials and fouling of cathodes constitute the main drawbacks associated with electrolytic processes. On the contrary, bioflocculation is an environmentally friendly dewatering method [12, 13], less energy consuming [14, 36], requires few minutes as running time, and can achieve high efficiency not only at lab scale as presented herein. Additionally, no greenhouse gases emitted during bioflocculation process, therefore it is worthy to be applied for harvesting algal biomass at industrial scale which has been recognized as the cornerstone challenge to microalgae industry referring to the high cost associated with microalgae biomass dewatering.

Different pilot-scale studies about microalgae harvesting have been reported [28, 37–41]. Pfeiffer and Rusch [40] have reported a harvesting efficiency of 90 % for *Chaetoceros muelleri* employing centrifugation, however it is well known that centrifugation is costing due to operational energy and capital cost thus centrifugation process is not suitable for the production of low value products such as biofuel. Knuckey et al. [28] have reported a flocculation efficiency ≥80 % while harvesting 10–1000 L cultures of different microalgae species by pH increment in the presence of a non-ionic polymer Magnafloc LT-25. Csordas and Wang [41] have reported a harvesting efficiency of 90 % while treating *Chlorella* sp. by foam fractionation. However the technology requires the use of chemicals (such as cetyl trimethylammonium bromide, CTAB) which may interfere with subsequent downstream processing of algal biomass harvested.

Innovative and effective microalgae harvesting/dewatering by various means of bioflocculation have been reported [7, 15–18]. Recently the mechanism governing microalgae bioflocculation utilizing bacterial bioflocculant was developed [19]. Lee et al. [36] have estimated the mixing energy and process cost for algal microbial flocculation during their study on energy requirements and economic analysis of a full-scale microbial flocculation system of microalgal harvesting. Authors proposed a full-scale microalgae harvesting system based on laboratory data, and found that mixing energy required for the bioflocculation was estimated to be 0.893 kWh/10^3^ kg of dry mass flocculated, with estimated overall process cost of $0.12/m^3^ of the culture medium flocculated, a process cost relatively low compared to the self-cleaning centrifuge, conventional flocculation, flotation with flocculant, and electro-flocculation [36]. The energy consumption during harvesting step for any technology used was estimated previously between 8.2 and 32 kWh/kg dry mass [42], hence the commercialization of algal biomass would be suitable only for high value products. If algal biomass is to be utilized for biofuel production, the harvesting energy should be less than 1.8 kWh/kg dry mass [43].

In attempt to harvest algal biomass at low cost (low energy consumption), Berrut et al. [39] studied the separation efficiency of a vacuum gas lift for microalgae harvesting. Authors found that the harvesting cost could be greatly reduced as the harvesting system would require energy between 0.16 and 3.37 kWh/kg dry mass, therefore algal biomass would be suitable for production and commercialization of low value products such as biofuel. Even though the vacuum gas lift was found effective for microalgae harvesting, large volumes of the harvest would need additional cost for maintenance such as refrigerators and possibly affect further downstream processes. The same observations were remarked by Knuckey et al. [28] during their study on production of microalgal concentrates by flocculation and their assessment as aquaculture feeds. Authors realized that large volumes about 40 L remained after induced pH flocculation of 1000 L. Nevertheless, based on laboratory data, Lee et al. [44] estimated the mixing energy and harvesting cost for a designed electro-flocculation plant for harvesting marine microalgae. Authors achieved an overall energy consumption of 0.092 kWh/m^3^ of culture medium treated and a total harvesting cost of $0.19/kg of the ash-free dry mass. However, only energy consumption, based on electro-flocculation separation and mechanical mixing, electrode dissolution and construction costs were considered in the study, while the electrode material cost and their regular replacement were neglected, which cause the most hindrance to electro-flocculation [45–47]. Recently, Selesu et al. [38] introduced a promising method for harvesting microalgae biomass by flocculation with a cheap and nontoxic flocculant (Tanfloc). Authors compared the cost of harvesting microalgae *Scenedesmus* sp. by flocculation utilizing chitosan and Tanfloc. The harvesting cost was relatively low for Tanfloc ($1.10/kg of dry mass) compared to chitosan ($10.00/kg of dry mass). However, mixing as one of the key factors for the success of flocculation was not considered in the study, apparently the harvesting cost could increase once the mixing energy is incorporated into the study.

Table 3 presents a summary of comparative energy requirement and cost analysis of different reported microalgae harvesting technologies. Electro-flocculation, microbial flocculation, flotation with vacuum gas lift, and TFF are in the same range of process cost (Table 3); however the long mixing time incurred during microbial flocculation increased the process cost, thus with the newly fast-developed bioflocculation herein presented, the process cost could be further reduced, thus placing bioflocculation at the forefront of the cheapest microalgae harvesting methods.

**Table 3:** Comparative energy and cost analysis of different microalgae harvesting technologies

Harvesting method	Algae species	Energy input (kWh/m^3^)	Cost ($/kg)	References
Microbial induced flocculation	*Pleurochrysis carterae*	0.45 × 10^−3^	0.24	[36]
Centrifugation	*Scenedesmus, Coelastrum proboscideum*	0.3–8	1.44–18	[55]
Flotation with vacuum gas lift	Not precised	0.8–1.69	0.022–0.44	[39]
Filtration with vacuum filter	*Scenedesmus, Chlorella proboscideum*	0.1–5.9	0.96–9	[55]
Electro-flocculation	*Tetraselmis* sp.	0.092	0.19	[44]
Flocculation with Tanfloc	*Scenedesmus* sp.	ND^a^	1.10	[38]
Flocculation with Zetag 7650 and Al_2_(SO_4_)_3_	*Tetraselmis suecica*	14.81	~1.8	[56]
Filtration with pressure filters	*Chlorella proboscideum*	0.5–0.88	0.8–2.2	[55]
Flocculation with chitosan	*Scenedesmus* sp.	ND	10.00	[38]
Tangential flow filtration (TFF)	*Tetraselmis* s*uecica*	2.06	~0.26	[56]

The energy consumption and cost data of microalgae harvesting are presented based on the culture medium (m^3^) treated and dry mass (kg) produced, respectively

^a^Not determined

Considering experimental data previously reported [7, 15–18], pilot-scale data herein presented and mixing energy requirement and process cost as estimated by Lee et al. [36], with many other advantages [12, 13], doubtlessly bioflocculation is a preferable choice for microalgae harvesting either for biofuel production or for feed and food purposes.

The results presented by Lee et al. [36, 44] are estimates for designed large-scale microalgae harvesting systems, therefore a full life-cycle assessment (LCA) study based on a pilot-scale or a full-scale data considering all input materials incurred for microalgae harvesting unit would be appropriate to provide more accurate information.

## Conclusion

Bioflocculation is an efficient, environmentally friendly, and cost-effective microalgae dewatering technique. This work examined the pilot-scale and in situ flocculation of the freshwater microalgae *D. brasiliensis* induced by the bioflocculant γ-PGA produced by *B. licheniformis* CGMCC 2876. Advantages of bioflocculation in situ include reduced process cost as no mixing device and separate flocculation tank are required. Moreover, the process time is shortened as there is no need to move the cultures from cultivation area to flocculation area. The method was found scalable achieving over 98 % efficiency at the scale of 200 L of microalgae cultures and at in situ flocculation of 50 L of cultures. Doubtlessly, the process can be industrialized. Furthermore, biochemical composition analysis of microalgae biomass revealed that bioflocculation technology is worthy to be applied for harvesting algal biomass to be used for biofuel production. Even though the process was found scalable, the design of the mixing apparatus and air flow rate are the key parameters for effective mixing, therefore further studies are necessary to develop a most favorable and well-designed mixing device as well as optimizing the air flow rate for in situ treatment. Furthermore, it is essential to carry out a life-cycle assessment based on pilot-scale or full-scale data generated by flocculation process applying microbial flocculant in order to have more accurate information on energy consumption and process cost. This will contribute more to further development and commercialization of microalgal-based products.

## Methods

### Microalgal strains and cultivation medium

The freshwater microalgae *D. brasiliensis* (collection number FACHB-1495) used in the current study was purchased from the Freshwater Algae Culture Collection at the Institute of Hydrobiology, CAS, Wuhan, China. Modified Bold 3 N medium was used for microalgae cultivation, consisting of (g/L): NaNO_3_, 0.750; CaCl_2_·2H_2_O, 0.025; MgSO_4_·7H_2_O, 0.057; K_2_HPO_4_, 0.0383; KH_2_PO_4_, 0.088; and NaCl, 0.025. Metal and vitamin solutions are as described previously by Berges and Franklin [48].

### Microalgae culture and bioflocculant production

**Fig. 5:**
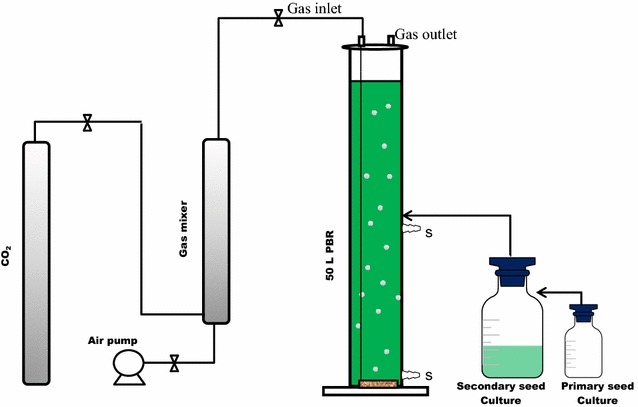
Microalgae cultivation system. The figure depicts a cultivation system applied for the 50 L column PBR (the primary seed culture was utilized to inoculate the second seed culture which was also utilized to inoculate the PBR culture, gas mixer helped to mix CO_2_ and air before they are feed into the PBR culture, *S* sample port)

The proposed pilot-scale scheme for microalgae cultivation and harvesting is described in Fig. 5. The culture of microalgae for primary and secondary seed cultures was operated according to the procedures described in our previous report [7]. The secondary pre-cultured microalgae (5 L) were inoculated into the 50 L photobioreactor (PBR) containing 45 L of the cultivation medium prepared with the tap water without sterilization; however, the culture growth rate was low compared to the cultures conducted with sterilized water possibly due to other microbes present in the unsterilized water culture. Cultivation conditions: 2.5 % CO_2_, aeration rate 0.06 vvm. The light (light intensity ~ 2400 μmol/m^2^·s) was continuously (24 h) provided by external light sources (14 W TL5 tungsten filament lamps, Philips Co., China) mounted on one side of the PBR for indoor cultivation, while the sun light was the sole light source for outdoor cultivation. The microalgae were harvested at the end of the exponential growth phase.

The bacteria *B. licheniformis* CGMCC 2876 isolated and identified by the Department of Chemical and Biochemical Engineering (Xiamen University) was used to produce the γ-PGA broth bioflocculant and the production process was operated as previously described by Xiong et al. [49]. Analysis of the flocculating activity of the bioflocculant and determination of the bioflocculant concentration were conducted following the procedures described in our previous report [19]. The flocculation cost could be greatly reduced by in situ treatment as no investment cost is needed for a separate flocculation tank and mixing device. Therefore it was imperative to examine in situ flocculability of the freshwater microalgae *D. brasiliensis*. After the cultivation period, the supply of CO_2_ was stopped; the pH value of microalgae cultures was adjusted to three using 1 M HCl. The bioflocculant (2.5 mL/L) was added to the cultures followed by mixing by air at aeration rate of 0.1 L/min for 2 min, and the flocs formed were allowed to settle under gravity. Samples were collected after 1, 5, 10, 15, and 20 min for flocculation efficiency analysis. The broth of *B. licheniformis* CGMCC 2876 was used as the bioflocculant without further purification processes.

### Determination of algal biomass concentration

The calibration curve expressed in Eq. (1) relating the dry cell weight (DCW) to the optical density values at the wavelength of 685 nm (OD_685nm_) was used to estimate the microalgae biomass concentration. The optical density values were determined utilizing the UV Spectrophotometer (UV-1780, SHIMADZU, Kyoto, Japan). The DCW was obtained by weighting the microalgae cells after washing two times with deionized water, followed by subsequent overnight drying till a constant weight was achieved in an oven at 80 °C.

1 $$y = 1.526x - 0.078$$

with $y$ the DCW (g/L) and *x* the OD685_nm_.

### Microalgae flocculation experiments

Flocculation experiments were carried out with 5, 50, and 200 L of microalgae cultures. After the distribution of microalgae cultures into flocculation vessels, the pH value of the culture was gradually adjusted to pH 3 using 1 M HCl, thereafter the bioflocculant was added (2.5 mL/L), followed by mixing at 200 rpm for 2 min, then the mixing was stopped to allow the flocs formed to settle at room temperature. The operating parameters for the pilot-scale were supposed to be the same as the optimum operating parameters reported at lab-scale [7], however the mixing capacity of the device used could not be adjusted below 200 rpm, thus only mixing at 200 rpm was possible and no slow mixing was applied. In situ flocculation was conducted in the same photobioreactor (50 L) used for microalgae cultivation. After the cultivation time, the CO_2_ was stopped and air was used for mixing. The flocculation efficiency (FE) was calculated according to Eq. (2):

2 $${\text{F}}.{\text{E }}\left( {\text{\%}} \right) = \frac{{{\text{OD}}_{0} - {\text{OD}}_{1} }}{{{\text{OD}}_{0} }} \times 100$$

where ${\text{OD}}_{0}$ and ${\text{OD}}_{1}$ are the OD685_nm_ values of the microalgal suspension before and after flocculation, respectively.

### Microalgae biomass characterization

Biomass characterization was conducted in order to examine the applicability of bioflocculation as a dewatering method for the recovery of different microalgal biomass biochemicals. The results will provide insight into the scalability of bioflocculation for the recovery of different microalgal biomass constituents vs. centrifugation widely applied in industry. Microalgae biomass harvested by either centrifugation at 6000 rpm for 10 min (Centrifuge, D-78532 Tuttlingen, ZENTRIFUGEN, Germany) or bioflocculation was freeze dried for 24 h at 3 kPa (Freeze dryer, FD-1000, EYELA, Japan) and stored at −4 °C before analysis. The biomass biochemicals recovered from both harvesting methods were compared through One-way ANOVA, using Fisher’s protected least significant difference (PLSD) test for pair-wise comparisons (OriginPro 8.6, OriginLab Corporation, USA).

### Determination of total carbohydrates

The colorimetric method (Phenol–Sulfuric Acid method) widely applied for the determination of total carbohydrate content in liquid solutions was employed in this study. Experiments were conducted according to the procedures described by Albalasmeh et al. [50] with modifications. Briefly, 10 mg of freeze-dried biomass was reconstituted in 10 mL of deionized water. 2 mL of reconstituted solution (carbohydrate solution) was mixed with 1 mL of phenol (5 % w/v) in a test tube. Subsequently, the mixture was rapidly reacted with 5 mL of concentrated sulfuric acid. After 10 min standing at room temperature, the tubes were vortexed for 1 min and then placed in water bath for 20 min at 30 °C for color development. Blanks were prepared in the same way as described above, and 2 mL of dH_2_O was used instead of carbohydrate solutions. After the color development, light absorption at the wavelength of 490 nm was recorded using UVS pectrophotometer (UV-1780, SHIMADZU, Kyoto, Japan). The total carbohydrates were determined referring to the standard curve (Eq.(3)) based on glucose (Additional file 1: Fig. S1a), phenol (5 % w/v in water), and glucose solution for the standard curve were prepared freshly before the experiments. All samples were prepared in triplicates.

3 $$y = 12.82x \pm 0.14$$

with $y$ the OD490_nm_ and *x* the glucose concentration (mg/L).

### Determination of total proteins

In order to estimate the total proteins, Bradford method invented by Bradford [51] was utilized in the present study. Bradford method is the most convenient, simple, faster, and more preferred method for protein quantification in solutions [52]. This method is known to be less subjected to interference by common reagents and non-protein components of samples like Lowry method [52]. Bradford protein assay protocol [53] (http://www.bio-protocol.org/e45) was followed with modifications. The protein solution was prepared by solubilizing 10 mg of freeze-dried biomass in 10 mL of dH_2_O. An aliquot of 0.1 mL of protein solution was reacted with 1 mL of the Bradford reagent, vortexed and incubated for 5 min at room temperature for color development. The blank was prepared by replacing the protein sample by dH_2_O. All samples were prepared in triplicates. The absorbance was measured at 595 nm wavelength utilizing a UV Spectrophotometer (UV-1780, SHIMADZU, Kyoto, Japan) and the total protein concentration was determined referring to the Bovine Serum Albumin **(**BSA) standard curve (Eq. (4)) (Additional file 1: Fig. S1b):

4 $$y = 4.45x \pm 0.11$$

with $y$ the OD595_nm_ and *x* the BSA concentration (mg/L).

### Determination of total lipids

In the current study, total lipids extraction was performed according to the method developed by Axelsson and Gentili [25] with modifications. Freeze-dried biomass (20 mg) was placed in pre-weighted centrifuge tube, and then 8 mL of chloroform–methanol (2:1 v/v) mixture was added. Algal biomass was suspended manually by shaking vigorously the centrifuge tubes for 1 min, and then 2 mL of 0.73 % NaCl aqueous solution was added. The mixture was centrifuged at 120 × 10^3^ rpm for 10 min (Centrifuge, himac CT15, HITACH, Japan) to allow phase separation. The upper phase was siphoned and the solvent was evaporated under a nitrogen stream. After the solvent was completely evaporated, lipid fractions were quantified gravimetrically by reweighting the centrifuge tube. The percentage of total lipid content was determined by dividing the weight of recovered lipids by the weight of the dried biomass. All experiments were conducted in triplicates.

## Additional file

10.1186/s13068-016-0458-5 Glucose and BSA standard curves. The standard curve describes glucose and BSA standard curves, (a) for total carbohydrates and (b) for total proteins quantification, respectively.

## Abbreviations

HPLC: high-performance liquid chromatography
HPAEC: high-performance anion exchange chromatography
GC: gas chromatography
γ-PGA: poly γ-glutamic acid
CTAB: cetyl trimethylammonium bromide
LCA: life-cycle assessment
PBR: photobioreactor
DCW: dry cell weight
BSA: bovine serum albumin
Fig.: figure
