# Supplementary material for: Harvesting of freshwater microalgae with microbial bioflocculant: a pilot-scale study
Source: Biotechnol Biofuels. 2016 Feb 27;9:47. doi: 10.1186/s13068-016-0458-5 (PMC4769512; doi:10.1186/s13068-016-0458-5)
Supplement: Supplementary file 1 — 10.1186/s13068-016-0458-5 Glucose and BSA standard curves. The standard curve describes glucose and BSA standard curves, (a) for total carbohydrates and (b) for total proteins quantification, respectively. [file 13068_2016_458_MOESM1_ESM.docx]

# Additional file

1. Glucose and BSA standard curves

Fig.S1. Standard curves: (a) for total carbohydrates and (b) for total proteins
